# Translation and psychometric validation of the Finnish version of the revised second victim experience and support tool: a pilot study

**DOI:** 10.1186/s12912-025-03396-z

**Published:** 2025-07-01

**Authors:** Sanu Mahat, Saija Koskiniemi, Helena Lehmusto, Santtu Mikkonen, Tiina Syyrilä, Marja Härkänen

**Affiliations:** 1 Department of Nursing Science, University of Eastern Finland, Yliopistoranta 1c, Kuopio, Finland; 2 Helsinki University Hospital Pharmacy, Jorvi Hospital, Turuntie 150, Espoo, Finland; 3 Faculty of Science, Forestry and Technology, Department of Environmental and Biological Sciences, University of Eastern Finland, Yliopistonranta 8, Kuopio, Finland; 4 Academy of Finland, Research Centre for Nursing Science and Social and Health Management, Kuopio University Hospital, Wellbeing Services County of North Savo, Kuopio, Finland

**Keywords:** Second victim, Medication error, Healthcare professionals, SVEST, Patient safety, Adverse events, Second victim syndrome

## Abstract

**Background:**

Unintentional healthcare harm can also affect involved healthcare professionals making them the second victims. Second victims are found to exhibit several physical and psychological symptoms which affect their personal, social and professional lives. The revised version of the second victim experience and support tool (SVEST-R) has been widely acknowledged given its use to assess second victim experiences among healthcare professionals.

**Aims:**

To translate the revised second victim experience and support tool into Finnish and test the psychometric properties and feasibility of the Finnish version (FI-SVEST-R).

**Design:**

A cross-sectional online survey was adopted. This study adheres to the STROBE reporting guidelines.

**Methods:**

The tool was translated into Finnish using forward and backward translation which was evaluated by the expert panel including its cultural appropriateness. An online survey was conducted among Finnish healthcare professionals in two different university hospitals between September 2022 and April 2023 to assess the comprehensibility of the questions, response options and feasibility of the tool. The confirmatory factor analysis was conducted for construct validity, and internal consistency was examined.

**Results:**

A total of *n* = 145 healthcare professionals participated in the study. Results showed a good model fit for the nine-factor structure with 35 items. Root mean squared error of approximation (0.067), comparative fit index (0.895), and Tucker-Lewis index values (0.880) suggested a good model fit. McDonald’s Omega ranged from 0.755 to 0.909, with Cronbach’s alpha ranging from 0.789 to 0.934, whereas factor loadings for all items ranged from 0.390 to 0.970.

**Conclusions:**

The Finnish version of the revised second victim experience and support tool (FI-SVEST-R) was proven to be a valid and reliable tool with strong psychometric properties during the pilot testing. Thus, it can be used to evaluate the extent of the second victim phenomenon and available support resources in Finnish healthcare. It could also be used to develop and evaluate the effectiveness of support programmes. However, due to the relatively low sample size and low factor loadings of a few items, further validation studies are required.

**Impact:**

FI-SVEST-R can provide an option for the quantitative measurement of the problem related to second victim distress and available support resources in Finland.

**Clinical trial number:**

Not applicable.

**Supplementary Information:**

The online version contains supplementary material available at 10.1186/s12912-025-03396-z.

## Background

In healthcare, errors are common and can result in various degrees of harm to patients. Patients are always the first victims of errors, and protecting patients after these events is a high priority in healthcare. However, this overlooks the healthcare professionals (HCPs) who might be equally affected by these events [1]. In modern healthcare, it is widely recognised that errors can also cause unintentional harm to the HCPs involved making them second victims. The term ‘second victim’ was first introduced by Albert Wu [2]. Wu first discussed this term in relation to the experiences of medical doctors in the aftermath of adverse events, but the definition was later expanded to include all HCPs [3]. The second victim phenomenon occurs when a critical incident has led to a serious safety event for the patient or workplace [4].

Second victim syndrome (SVS) has been defined as: ‘the HCPs who commit an error and are traumatised by the event manifesting psychological (shame, guilt, anxiety, depression or grief), cognitive (burnout, compassion dissatisfaction, stress) and/or physical reactions that have a personal negative impact’ [1, 4]. Negative emotions after patient safety incidents expressed in incident reports have been found to include fear, disturbance, sadness, and guilt among HCPs [5]. Being a second victim might cause different symptoms and affect the well-being of HCPs, reducing job confidence and satisfaction and increasing anxiety, stress, and sleep disorders [6]. This in turn might increase the risk of absenteeism and turnover intention [7]. In many cases, SVS experiences are long-lasting and can sometimes lead to harmful self-care behaviours, such as substance abuse [6].

The second victim phenomenon is quite common. Approximately 9-50% of HCPs have experienced SVS at least once in their lifetime, based on previous research [4]. However, the concept remains relatively unknown among HCPs [4], and there is a substantial variation across organizations worldwide in terms of how SVS is treated and how HCPs are supported. Measuring the second victim experiences of HCPs is essential for providing appropriate support and reducing the stigma associated with the phenomenon.

The original Second Victim Experience and Support Tool (SVEST) was developed in the United States in 2017 [8]. The revised version of the tool SVEST (SVEST-R) was developed based on the original SVEST, literature and expert suggestions [9]. Multiple translations of SVEST and SVEST-R have been completed to date (Appendix 1). The majority of these translations have been conducted using the original SVEST. The SVEST has been translated into Danish [7], Italian [10, 11], Korean [12], Mandarin Chinese [6], Persian [13, 14], Portuguese [15], Spanish [16], and Turkish [17]. The SVEST-R has been translated into German [4], Malay [18], and Thai [19]. Previous qualitative studies conducted among Finnish HCPs have highlighted the impact of the second victim phenomenon and the existing support strategies in Finland [20, 21]. This demands a quantitative tool to measure the extent of this problem in Finland, which could be SVEST-R. However, this globally accepted tool to measure the second victim phenomenon and perception of support has not been translated and validated for use in the Finnish context. Therefore, this is the first study translating and examining the construct validity and internal consistency of the SVEST-R in Finland.

## Methods

### Aims

This study aims to translate SVEST-R into the Finnish language and test the psychometric properties and feasibility of the Finnish version of SVEST-R (FI-SVEST-R).

### Study design and participants

This pilot cross-sectional survey study was conducted in two different university hospitals in Finland between September 2022 and April 2023. A total of 5000 nurses and physicians worked in those hospitals. Study participants were HCPs (nurses and physicians) from those hospitals who had experienced medication errors (MEs) in their working careers and had felt negative emotions after committing MEs. Due to the lack of individual-level tracking, it was not possible to determine how many of those HCPs received or read the survey invitation. A total of 498 HCPs opened the survey links, of which 166 (33.33%) nurses and physicians participated. The Strengthening the Reporting of Observational studies in Epidemiology (STROBE) guidelines (supplementary file) were used to report the study [22].

### SVEST-R instrument

Burlison and colleagues developed the first version of the SVEST to assist healthcare institutions in recognising the necessity and monitoring the implementation of support resources for second victims [8]. The original SVEST includes 29 items comprising seven factors and two outcome variables. All items are closed-ended statements ranked on a 5-point Likert scale ranging from 1 (strongly disagree) to 5 (strongly agree). High scores denote a high prevalence of second victim distress, an increased sense of inadequate support, and a high frequency of unfavourable work outcomes. The seven factors are psychological distress (four items), physical distress (four items), colleague support (four items), supervisor support (four items), institutional support (three items), non-work-related support (two items), and professional self-efficacy (four items). The two outcome variables are ‘turnover intentions’ (two items) and ‘absenteeism’ (two items).

The original SVEST was later revised into a 35-item SVEST-R questionnaire that omitted the non-work-related-support dimension and added a positive outcome dimension; resilience [9]. The SVEST-R demonstrated a good construct validity based on confirmatory factor analysis (CFA) (chi-square test x^2^ = 1555, degree of freedom [DOF] = 524, root mean square error of approximation [RMSEA] = 0.079, comparative fit index [CFI] = 0.821, and standardised root mean squared residual [SRMR] = 0.091). Cronbach’s alpha ranged from 0.66 for ‘colleague support’ to 0.86 for ‘physical distress’, whereas factor loadings of all items ranged from 0.42 to 0.92.

### Translation and cultural adaptation of the tool

#### Step 1: Preparation

The sixth author emailed the original author of the SVEST to seek permission to translate the tool into Finnish. After receiving authorisation from Professor James Hoffman in April 2022, the authors adhered to a multi-step process in line with the World Health Organization (WHO) guidelines for translation and cultural adaptation [23].

#### Step 2: forward translation

Two bilingual researchers (the third and fifth authors of this paper) working in nursing and patient safety translated the tool from English to Finnish. After the translation, the last author, who is also an expert in patient safety, assessed the consistency and adaptability of the Finnish context between the translated and original versions.

#### Step 3: cultural appropriateness testing

This was conducted by a team of bilingual researchers, including two working in patient safety and two original translators. In this step, the researchers evaluated and compared the Finnish translations to English to select more culturally appropriate words with similar meanings. Necessary revisions were made after team discussion.

#### Step 4: back translation

A professional translator who is a native Finnish speaker and an expert in medical English back-translated the full revised and translated version of the instrument into English and checked for the appropriateness of the terminologies used.

#### Step 5: revision of back translation by bilingual expert panel members

The third author of the paper contacted 15 expert panel members, eight of whom agreed to review the translated version of the SVEST-R. The eight expert panel members consisted of three pharmacists, one physician and four nurse researchers known for their work in the field of patient safety. A 3-point numerical scale (1 = not suitable, 2 = needs more detail, 3 = suitable) was used to evaluate the accuracy of the translation. The suitability of the statements was further evaluated using a 4-point numerical scale (0 = not suitable at all, 1 = not suitable, 2 = suitable, and 3 = very suitable). Following the recommendations of expert panel members, certain wordings and sentence structures were revised to better align with Finnish culture. One of the items (item 24) was required to be marked as a reverse-coded item to ensure the comparability with the original SVEST-R, as the direction of the statement changed while translating it to Finnish, even though the meaning remained consistent.

### Data collection

An online survey link was created using Webropol software (V3.0; Webropol Oy, Helsinki, Finland) and distributed via email along with information about the research. This was facilitated through contact personnel in both university hospitals. The data were collected during two different periods, September 2022-October 2022 and January 2023-April 2023. Multiple reminders were sent to both hospitals.

### Data analysis

#### Confirmatory factor analysis

CFA aims to confirm the existing model or modify the model to improve the model fit (Schreiber et al., 2006). SPSS 29 and IBM SPSS Amos 29 were used for the analysis. The CFA was conducted through the following seven steps, adopted from Meyers et al., [24], Schreiber et al., [25] and Kääriäinen et al. [26]:

##### Data Preparation

The data underwent thorough screening to ensure adequacy for CFA. This involved checking for a sample size of at least three times the number of variables and a minimum of 100 respondents and examining the nature of missing data. Missingness was evaluated to determine whether it adhered to the missing completely at random (MCAR) assumptions. This assessment helped guide the appropriate handling of missing data in subsequent analyses.

##### Model specification

A carefully crafted model, rooted in prior CFA, was specified. It was crucial to confirm that the current dataset differed from any datasets used in previous analyses. The model delineated latent dependent variables (factors), independent variables (items), and error terms, with careful consideration given to the theoretical underpinnings guiding the model.

##### Model identification

The model underwent rigorous identification procedures to ensure it was statistically estimable and possessed a positive number of degrees of freedom (df). This involved examining the presence of linear dependencies among the model parameters and ensuring that the model was identifiable.

##### Model Estimation

Utilising the maximum likelihood (ML) estimation method, the model’s parameters were estimated. Standardised regression coefficients were employed for parameter estimation, providing insights into the strength and direction of relationships between variables.

##### Model evaluation

Correlations between items within the anticipated scale were scrutinised, ensuring the coherence of the measurement model. Standardised regression weights were examined to verify the statistical significance of all items. Model fit was assessed using various indices (target value in the brackets), including the comparative fit index (CFI > 0.950), Tucker-Lewis index (TLI > 0.950), and RMSEA (< 0.100). These indices provided nuanced insights into the overall fit of the model to the data, with specific target values indicating acceptable fit levels (Hu & Bentler, 1999; Schreiber et al., 2006; Jackson et al., 2009; Brown, 2015).

##### Model modification

Modification indices (MI) were leveraged to identify potential areas for model improvement. These indices flagged areas where adding or removing specific paths or covariances could substantially enhance model fit. By iteratively assessing MI and implementing targeted modifications, the model’s fit could be optimised. This process involved carefully considering the theoretical implications of each modification to ensure they were theoretically justifiable and aligned with the study’s objectives.

##### Results reporting

**Fig. 1:**
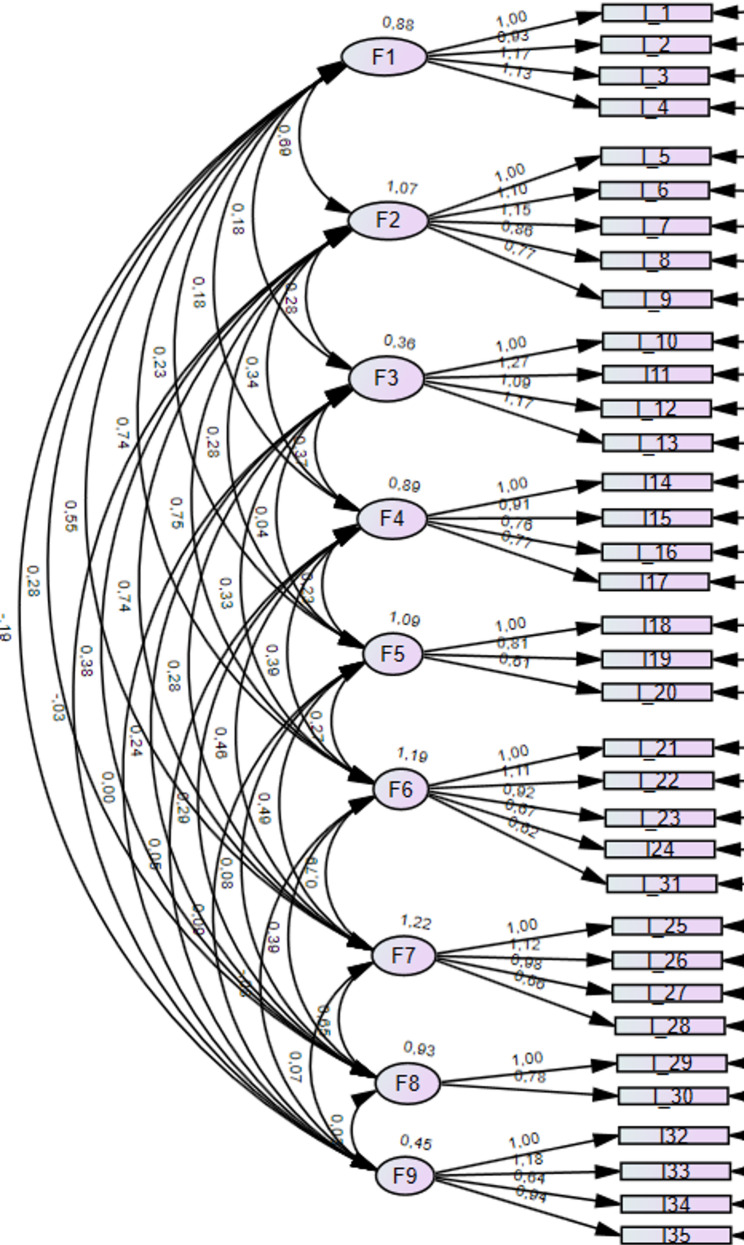
The modified FI-SVEST-R model output from AMOS with nine factors and 35 items

Results were carefully reported, detailing regression weights, standardized regression weights, standard deviations, p-values, and covariances. The confirmation of model fit through CFI, TLI, and RMSEA indices was emphasized. Additionally, the factor structure was visually presented (Fig. 1), providing a clear overview of the model’s underlying relationships and constructs.

## Results

### Characteristics of the sample

The number of included respondents was *n* = 145. Most of the respondents were females (92.4%) and nurses (93.8%), with only 6.2% of respondents being physicians. One-third (31.7%) of respondents had work experience of more than 21 years, and 22.1% of respondents had work experience of 1–5 years. The respondents were evenly distributed across all age groups (24.8-28.9%).

### Construct validity using CFA

The original raw data included 166 responses, 21 of which had missing values. Little’s MCAR test indicated that the missingness was completely random (chi-square = 10.451, DF = 17, sig. =0.884). These 21 responses were removed before the final analysis with Amos. The included data for analysis had no missing values, and all variables were correlated. The construct validity for the pilot testing of FI-SVEST-R was assessed using CFA with a sample of 145 HCPs. The sample size met the minimum requirements (*n* = 145/ 42 variables > 3) and over 10 variables loaded above 0.40 (41 variables out of 42). The model was based on the previously validated SVEST-R scale [9]; thus, CFA was deemed appropriate. The model was identified by a df of 490. The default ML application results of the standardised regression coefficients for parameter estimations are presented in Table 1. Covariance estimates ranged between − 0.193 and 0.788 (Appendix 3). Firstly, CFA included the support desirability part of SVEST-R; however, after careful consideration, it was excluded from CFA due to its conceptual difference from other constructs. As other constructs focused on actual experiences of HCPs, this part represents the preferences or desires of HCPs towards potential support options. Thus, the second model was completed with nine factors, excluding the support desirability part and its seven items.

**Table 1:** Parameter estimates for 35-item FI-SVEST-R (*n* = 145)

Item vs. Factor	Standardized Regression weight Estimate (Factor Loadings)	Regression weight Estimate	S.E.	C.*R*.	*p*
**Psychological distress**					
1. I have experienced embarrassment from these instances.	0.772	1.000	-	-	-
2. My involvement in these types of instances has made me fearful of future occurrences.	0.725	0.926	0.104	8.895	< 0.001
3. My experiences have made me feel miserable.	0.856	1.173	0.109	10.736	< 0.001
4. I feel deep remorse/guilt for my past involvements in these types of events.	0.811	1.130	0.112	10.116	< 0.001
**Physical distress**					
5. The mental weight of my experience is exhausting.	0.812	1.000	-	-	-
6. My experience with these occurrences can make it hard to sleep regularly.	0.874	1.097	0.089	12.309	< 0.001
7. The stress from these situations has made me feel queasy or nauseous.	0.860	1.146	0.095	12.022	< 0.001
8. Thinking about these situations can make it difficult to have an appetite.	0.768	0.858	0.083	10.289	< 0.001
9. I have had bad dreams as a result of these situations.	0.638	0.774	0.095	8.126	< 0.001
**Colleague support**					
10. My colleagues can be indifferent to the impact these situations have had on me.	0.567	1.000	-	-	-
11. My colleagues help me feel that I am still a good healthcare provider despite any mistakes I have made. *	0.763	1.265	0.192	6.589	< 0.001
12. My colleagues no longer trust me	0.843	1.089	0.157	6.935	< 0.001
13. My professional reputation has been damaged because of these situations.	0.818	1.170	0.171	6.839	< 0.001
**Supervisor support**					
14. I feel that my supervisor treats me appropriately after these occasions. *	0.923	1.000	-	-	-
15. My supervisor’s responses are fair. *	0.834	0.909	0.070	13.030	< 0.001
6. My supervisor blames individuals	0.637	0.764	0.089	8.634	< 0.001
17. I feel that my supervisor evaluates these situations in a manner that considers the complexity of patient care practices. *	0.706	0.765	0.076	10.017	< 0.001
**Institutional support**					
18. My organization understands that those involved may need help to process and resolve any effects they may have on care providers. *	0.865	1.000	-	-	-
19. My organization offers a variety of resources to help get me over the effects of involvement with these instances. *	0.831	0.814	0.090	9.080	< 0.001
20. Concern for the well-being of those involved in these situations is not strong at my organization.	0.580	0.613	0.090	6.803	< 0.001
**Professional self-efficacy**					
21. Following my involvement, I experienced feelings of inadequacy regarding my patient care abilities.	0.871	1.000	-	-	-
22. My experience makes me wonder if I am not really a good healthcare provider.	0.883	1.105	0.078	14.151	< 0.001
23. After my experience, I became afraid to attempt difficult or high-risk procedures.	0.834	0.920	0.072	12.835	< 0.001
24. These situations have negatively affected my performance at work.*	0.606	0.672	0.084	7.982	< 0.001
*31. When I am at work*,* I am distracted and not 100% present because of my involvement in these situations.*	0.577	0.623	0.083	7.493	< 0.001
**Turnover intentions**					
25. My experience with these events has led to a desire to take a position outside of patient care.	0.901	1.000	-	-	-
26. Sometimes, the stress from being involved with these situations makes me want to quit my job.	0.913	1.118	0.067	16.620	< 0.001
27. I have started to ask around about other job opportunities.	0.794	0.984	0.076	12.909	< 0.001
28. I plan to leave my job in the next 6 months because of my experience with these events.	0.813	0.664	0.052	12.837	< 0.001
**Absenteeism**					
29. My experience with an adverse patient event or error has resulted in me taking a mental health day.	0.970	1.000	-	-	-
30. I have taken time off after one of these instances occurs.	0.858	0.784	0.066	11.947	< 0.001
**Resilience**					
32. Because of these situations, I have become more attentive to my work. *	0.826	1.000	-	-	-
33. These situations have caused me to improve the quality of my care. *	0.856	1.184	0.135	8.792	< 0.001
34. My experience with an adverse patient event or error has resulted in positive changes in procedures or care on our unit. *	0.390	0.639	0.146	4.382	< 0.001
35. I have grown as a professional as a result of an adverse patient event or error. *	0.600	0.936	0.134	6.986	< 0.001

Abbreviations: SE, standard error, CR, composite reliability, FI-SVEST-R, Finnish version of the revised second victim experience and support tool, * refers to reverse coded items

In order to determine the best model fit, some structural variations were tested. All nine factors with 35 items following the original SVEST-R were tested, which showed low factor loadings for one item (Item 34: 0.390). Despite this, the overall model fit indices were within acceptable thresholds (CFI: 0.884; TLI: 0.808; RMSEA: 0.079). As some of the items showed covariance, slight modifications were made based on the modification indices in model 2 while retaining the same structure, which slightly improved model fit. Based on the modification indices, we relocated one item to a different factor, which improved the model fit (CFI: 0.895, TLI: 0.880, RMSEA: 0.067). We also tested a fourth model, where we removed the item with the lowest factor loading. This slightly improved model fit (CFI from 0.895 to 0.901) with a minimal difference of 0.006. However, to maintain the integrity of the original scale, we chose to retain item 34 and finalised Model 3 as our final model, as the overall fit remained within the acceptable limits (Table 2).

**Table 2:** Model fit indices for FI-SVEST-R compared to original SVEST-R (*n* = 145)

Parameters	Model 1	Model 2	Model 3 (final model)	Model 4	Original SVEST-*R*
CFI	0.853	0.893	0.895	0.901	0.821
RMSEA	0.079	0.068	0.067	0.067	0.079
TLI	0.847	0.878	0.880	0.887	-

Therefore, for the FI-SVEST-R scale, model 3, the nine-factor model was approved with 9 factors, 35 items and 35 error terms, forming a total of 79 variables. In this final model, regression weights ranged from 0.613 to 1.265, confirming the statistical significance of all items (< 0.001). The standardized regression weights (= factor loadings) ranged acceptably between 0.390 and 0.970, with all other items above 0.56 except one (Table 1). The baseline comparison values (CFI: 0.895; TLI: 0.880) demonstrated a good model fit, with CFI slightly below the cutoff of 0.90 with RMSEA of 0.067 indicating the good fit of the model.

### Descriptive statistics and internal consistencies

Table 3 presents internal consistencies which was assessed using both Cronbach’s alpha and McDonald’s omega and descriptive statistics for nine factors. Cronbach’s alpha was greater than 0.789, whereas McDonald’s Omega was greater than 0.755 for all factors.

**Table 3:** Means, sds, and reliability scores for FI-SVEST-R factors (*n* = 145)

Variables	Means	SD	McDonald’s Omega	Cronbach alpha	Items (*n*)
1. Psychological distress	3.32	1.06	0.871	0.934	4
2. Physical distress	2.67	1.11	0.897	0.868	5
3. Colleague support	2.47	0.41	0.785	0.891	4
4. Supervisor support	3.34	0.55	0.860	0.817	4
5. Institutional support	2.68	0.58	0.807	0.857	3
6. Professional self-efficacy	2.79	0.74	0.873	0.864	5
7. Turnover intentions	2.25	1.08	0.909	0.873	4
8. Absenteeism	1.76	0.86	N/A	0.905	2
9. Resilience	3.71	0.75	0.755	0.789	4

Abbreviations: SD, standard deviation; N/A: not applicable as McDonald’s omega could not be calculated for factors with less than 3 items

## Discussion

This pilot study translated the revised version of SVEST (SVEST-R) into Finnish (FI-SVEST-R) and tested its psychometric properties. All 35 items of the SVEST-R [9] were translated into Finnish following WHO guidelines [23]. The translation process did not reveal uncertainty between the FI-SVEST-R and the SVEST-R. The expert panel assessed that the FI-SVEST-R language and suitability to the Finnish healthcare context were adequate without major changes.

The SVEST-R is designed to evaluate second victim experiences among all HCPs [9]. This study’s data were collected from two university hospitals in Finland, with the majority of respondents having a nursing background, as with the validation processes of C-SVEST (Zhang et al., 2021) and P-SVEST [14]. The second victim phenomenon affects all HCPs, and most translations of SVEST or SVEST-R have been tested by other HCPs, including nurses [4, 7, 10, 17, 18]. 

In addition to Cronbach’s alpha, this study also assessed internal consistency using McDonald’s omega coefficient. Although the use of McDonald’s omega is sound from a methodological point of view [27], it has not been used in previous cross-cultural translation and validation of the SVEST and SVEST-R instruments. The overall Cronbach’s α calculated for FI-SVEST-R was 0.933, which is more than the overall Cronbach’s α (0.844) for G-SVEST-R [4]. In the original SVEST-R [9], Cronbach’s alpha ranged from 0.66 to 0.86, and in this study, it ranged from 0.789 to 0.934. The lowest score was for the ‘Resilience’ dimension in the FI-SVEST-R and the ‘colleague support’ dimension in the SVEST-R [9].

Three models were tested during the analysis. Model 1, including the support desirability part, which, after careful statistical and theoretical considerations, was removed from the CFA model. The second model was tested with nine factors and 35 items, like the original SVEST-R. Another model was also tested where one item with poor factor loading was deleted, resulting in a slight increase in CFI, with TLI and RMSEA achieving insignificant changes. Even though the factor loading for this item fell below the recommended threshold, it was retained due to its theoretical relevance and alignment with the original SVEST-R, acknowledging the fact that a small number of study samples could have influenced this relatively poorer factor loading. As this was a pilot study, structural consistency was prioritised. Future studies with more diverse samples should be conducted to assess the performance of this item.

The FI-SVEST-R scale, comprising nine factors and 35 items, was validated through CFA, indicating a good model fit measured by a CFI of 0.895 and a TLI of 0.882 and having a good fit with RMSEA of 0.067. This performance is notably superior to the original version of the SVEST-R (Appendix 2). The model fit of the FI-SVEST-R is slightly lower than that of the Malaysian version, M-SVEST-R [18], with a slight difference in values for CFI (0.946), TLI (0.935), and RMSEA (0.051). However, the M-SVEST-R included only seven factors and 32 items, compromising the comparison between these scales. As the Malaysian model showed an unsatisfactory fit with nine factors and 35 items (CFI: 0.888; TLI: 0.866; RMSEA: 0.069), they combined the ‘Psychological and Physical distress’, ‘Turnover intention’ and ‘Absenteeism’ factors and deleted three items. However, in 21 items of the remaining 32 items in the M-SVEST-R, the factor loadings for the FI-SVEST-R were higher, indicating that the FI-SVEST-R is a suitable solution in Finland.

Both the Finnish and original SVEST-R scales had the same factors and items, except that in the Finnish version, one item from the ‘Absenteeism’ factor was moved to the ‘Professional self-efficacy’ factor. As a result, the model fit improved. Standardised regression weights for FI-SVEST-R were higher in 28 of 35 items than in the original SVEST-R. The seven items with higher standardised regression weights in the original version were regarding bad dreams (FI-SVEST-R: 0.638 / SVEST-R: 0.68), supervisor evaluation regarding complexity of patient care (0.706/ 0.66), concern for the wellbeing of the involved persons in the organisation (0.580/0.76), negative impact on performance (0.606/0.75), taking time off (0.858/0.89), positive changes in procedures (0.390/0.599) and growing as a professional (0.600/0.79). The Thai-SVEST-R [19] has retained the same factors and items as the original SVEST-R, as well as slightly better model fit measures (CFI: 0.91; RMSEA: 0.04) and factor loadings of 20 items of 35, when compared to both SVEST-R and FI-SVEST-R.

Based on the model fit comparison results between the SVEST-R, FI-SVEST-R, M-SVEST-R, and Thai-SVEST-R models, the FI-SVEST-R shows a strong consensus of factors and items regarding model fit and factor loading measures (Table 4). In this study, the model was retained to facilitate result comparison with previous models. The model fit of the German version G-SVEST-R [4] cannot be compared as CFA measures were not reported. The other 11 existing SVEST scales are not revised R-versions (Appendix 2). Thus, they are not comparable with the R-version measures.

**Table 4:** Comparison between factors, items, internal consistencies and model fit indices between international SVEST-R translations and validations

Translated version of SVEST-*R*	Number of factors	Number of items	Cronbach alpha ranges	Model fit indices		
				CFI	TLI	RMSEA
SVEST-R	9	35	0.66–0.86	0.821	-	0.079
FI-SVEST-R	9	35	0.789–0.938	0.895	0.882	0.067
M-SVEST-R	7	31	-	0.946	0.935	0.051
G-SVEST-R	11	42	Total scale: 0.884	-	-	-
Thai-SVEST-R	9	35	0.73–0.92	0.91	-	0.04

Note: For M-SVEST-R, Raykov’s rho was calculated instead of Cronbach’s alpha and for the G-SVEST-R, model fit was assessed using Barlett’s sphericity test

Similar to the findings of previous cross-cultural validations of SVEST and SVEST-R, this study also found variability in factor loadings and item performances, raising concerns regarding the conceptual clarity of the tool. More specifically, the SVEST-R instrument combines items reflecting different factors of the second victim experience: antecedents (involvement in adverse events), consequences of it (emotional distress factors, and work-related impacts), and coping or responses (perceived support and resilience). Combining both distress-related and resilience-related constructs into a single tool, though it sounds comprehensive, might compromise the theoretical integrity of the scale. Even though this study retained all factors and items to ensure comparability, there is a need to critically reflect on the conceptual structure of SVEST-R for better clarity. Future research should consider separating these constructs for a more theoretically coherent model.

### Strengths and limitations

This study posed a few limitations. First, the data size was relatively small, even though multiple reminders were sent. While our sample size meets the 3:1 participant-to-item ratio commonly cited for pilot studies, this limits the model stability and generalizability, as a minimum of 200 samples or at least a 5:1 participant-to-item ratio is recommended for CFA. Therefore, the current findings should be interpreted as preliminary, warranting further validation. Second, when compared with the majority of previous studies involving only nurses, this study also had physicians as study samples. However, only a small portion of respondents were physicians, with more than 90% being females and nurses. This might have caused an overrepresentation of nurses and an underrepresentation of physicians and other HCPs. In addition, no other HCPs were studied who might have similar experiences in the data. Thus, the findings from this study might not fully hold the perspectives of other underrepresented HCPs. Also, findings from previous studies conducted on SVP has highlighted the influence of gender and professional role in experience intense negative emotions aftermath adverse events ([28]. These limitations warrant a future validation study to be conducted in the Finnish context for a more balanced representation of gender and professional groups of HCPs. All respondents worked in hospital settings, primarily in specialised healthcare, demonstrating a lack of perspectives from HCPs working in other settings. The measures of CFI, TLI and RMSEA were reported, facilitating comparison of the model fit with other SVEST-R studies (Table 4).

## Conclusions

The FI-SVEST-R tool is valid and reliable for evaluating the SVS, distress, and need for support in a Finnish healthcare context. The model fit results are competitive with previous SVEST-R studies. This tool could be utilised to advance the development and assessment of support programmes. It is important to continue measuring the second victim experiences of HCPs. This enables the provision of appropriate support and may reduce the stigma associated with the phenomenon. However, due to the limited sample size in this pilot testing, a validation study on the FI-SVEST-R is recommended.

## Electronic supplementary material

Below is the link to the electronic supplementary material.

Supplementary Material 1

Supplementary Material 2

Supplementary Material 3

## Data Availability

The data that support the findings of this study are not publicly available. However, it will be made available from the authors upon reasonable request.

## Abbreviations

ME: Medication Error
HCP: Health Care Professionals
SVS: Second Victim Syndrome
SVEST: Second Victim Experience and Support Tool
SVEST-R: Second Victim Experience and Support Tool-Revised
FI-SVEST-R: Finnish version of Second Victim Experience and Support Tool-Revised
CFA: Confirmatory Factor Analysis
DOF: Degree of Freedom
RMSEA: Root Mean Square of Approximation
CFI: Comparative Fit Index
SRMR: Standardized Root Mean Square Residual
WHO: World Health Organisation
MCAR: Missing Completely At Random
ML: Maximum Likelihood
TLI: Tucker Lewis Index
MI: Modification Indices
