# Supplementary material for: Translation and psychometric validation of the Finnish version of the revised second victim experience and support tool: a pilot study
Source: BMC Nurs. 2025 Jul 1;24:744. doi: 10.1186/s12912-025-03396-z (PMC12211338; doi:10.1186/s12912-025-03396-z)
Supplement: Supplementary file 2 — Supplementary Material 2 [file 12912_2025_3396_MOESM2_ESM.docx]

**Appendix 1.** Translation and psychometric validation of the original and translated SVEST scales.

| **Authors**  **(year),**  **country,**  **the name of the scale** | **Translation**  (into which language)  Method  1. Forward translation by  - Evaluated by  2. Back translation by  - Evaluated by  = Result | **Psychometric validation** | | | | | | **The result/**  **recommendations** |
| --- | --- | --- | --- | --- | --- | --- | --- | --- |
|  |  | **Face validity**  Method, participants (number)  = Result | **Content validity**  Participants (number)  = Result | **Pilot test**  Participants (number)  = Result | **The data**  Participants  (number)  = The total  Participants  working area | **Construct validity**  Methods  = Results | **1. Internal consistency**  Methods  = Results  **2. Construct**  **reliability**  Methods  = Results  **3. Convergent and divergent validity**  Methods  = Results  **4. Multivariate** **normality**  Methods  = Results |  |
| Brunelli, M.V., Estrada, S., Celano, C. (2021), Argentina,  The Argentinian version of the SVEST | (Spanish)  Mapi Research Institute model  1. Two native and bilingual persons independently, then merged into a single translation  2. Two additional experts  = One item was added into the dimension 4 due to translation challenges | Interviews, HCPs^1^ (5)  = Not any difficulties in understanding; the new item in dimension 4 was seen as necessary | Not reported | Not conducted | Nursing professionals:  nurses and nurses  in training  (169)  = 169  Different areas  of nursing care | EFA^2^ fit was assessed using The Bartlett sphericity test, and KMO3 varimax rotation was applied. A correlation >0.40 was accepted, 7-factor model.  = p=0.000, KMO^3^ 0.74 | 1. Cronbach’s alpha  = At first, alpha ranged from 0.33 to 0.85. After removing three items (from dimensions Colleague support, Supervisor support and Institutional support):  The total scale alpha 0.805.  Cronbach’s alpha ranges between dimensions from 0.56 to 0.85.  The lowest alpha in dimension Colleagues support (0.56). | The Argentinian version of the SVEST scale includes 7 factors with a total of 26 items.  Three items were removed from dimensions 3, 4 and 5 to obtain satisfactory reliability levels. In future research, the elimination of those three items (“My co-workers were indifferent to what happened”, “My supervisor blamed all team members”, and “Worrying for the welfare of the  people involved in this type of incidents is not characteristic of  my organization or hospital”) need to be evaluated. |
| Burlison, J., Scott, S.D., Browne, E.K., Thompson, S.G., & Hoffman, J.M. (2017),  USA,  The original SVEST | The original scale is in American English and developed based on the literature. | Not reported | Nurses (3), physicians (3), and pharmacists (3) participate in a content validity assessment exercise.  = 78% inter-rater agreement | Not conducted. | Nurses (124), pharmacists (24), attending/staff physicians (24), management (23), technicians (15),  and others (71)  = 281  Specialised  paediatric hospital | CFA^4^, the fit was assessed using the chi-square test, RMSEA^5^, and CFI^6^. A convention loading level >0.40 was accepted, 7-factor models were tested.  = After removing one item:  χ2 = 566.06,  df = 254, p < 0.01, CFI^6^ 0.910, RMSEA^5^ 0.066 | 1. Cronbach’s alpha  = The total scale alpha *not reported*. Cronbach’s alpha ranges between dimensions from 0.61 to 0.88. The lowest alpha in dimension Colleague support (0.61) and Institutional support (0.64). | The original SVEST includes 7 factors with a total of 29 items and 2 outcome variables. |
| De Sordi, L.P., Lourenção, D.C. de A., Gallasch, C.H., & Baptista, P.C. P. (2022),  Brazil,  BR-SVEST | (Portuguese)  *Method not reported.*  1. Three independent, bilingual translators, then merged into a single translation  2. Another independent translator  - The research team  - The equivalence was evaluated by nurses, a physician, a psychologist, and a Bachelor of Languages (7 experts)  = Agreement rate 88.7% | Not reported | Patient safety and work health experts (7)  = CVR^7^ of 31 items (86%) >0.741 and of 5 items (14%) <0.741. CVR^7^ ranges from 0.429 to 1. Items with low CVR^7^ were improved based on experts’ suggestions. | Nursing and medical staff, nutritionists, nursing technicians, pharmacists, physical therapists, psychologists, and speech therapists (31) | Not conducted | Not reported | Not reported | BR-SVEST includes nine factors with a total of 36 items. |
| Kamaruzaman, A.Z.M., Ibrahim, M.I., Mokhtar, A.M., Zain, M.M., Satiman, S.N., & Yaacob, N.M. (2022), Malaysia,  M-SVEST-R | (Malay)  Wild et al.’s (2005) guidelines  1. Two independent translators, then merged into a single translation  2. Two translators  - Cognitive debriefing of translation by medical officers (5) and nurses (5)  = A few difficult words changed | Patient safety, hospital quality and management, teaching, clinical care, and counselling experts (9)  = Some minor adjustments | Patient safety, hospital quality and management, teaching, clinical care, and counselling experts (9)  = I-CVI^8^ and S-CVI^9^ both >0.8 | Medical officers, nurses, and assistant  medical officers (30)  =Minor changes to the questionnaire and related video | Nurses (326), medical officers (17), house officers (5), assistant medical officers (2)  = 350  Anesthesiology  and critical care, paediatrics, surgery, internal medicine, obstetrics and gynaecology, orthopedics and others | CFA^4^, fit was assessed using the chi-square test, RMSEA^5^, CFI^6^, SRMR^10^, AIC^11^, BIC^12^, TLI^13^. A factor loading > 0.40 was accepted, 9 and 7-factor models were tested.  = Two new combined factors were created: distress (a combination of psychological and physical distress) and negative outcomes  (a combination of turnover intention and absenteeism). Three items were removed based on factor loading <0.41.  χ2 = 797, p < 0.0001, RMSEA^5^ 0.051, CFI^6^ 0.946, SRMR^10^ 0.055, and TLI^13^ 0.935 | 1. Raykov’s rho coefficient  = The total rho scale 0.83. Ranges between dimensions 0.68-0.93. The lowest rho in dimension Institutional (0.68). | M-SVEST-R includes 7 factors with a total of 32 items. The questionnaire is a valid and reliable scale. |
| Kim, E-M., Kim, S-A., Lee, J-R-. Burlison, J.D., & Geum, E. (2020),  South Korea,  K-SVEST | (Korean)  WHO’s guidelines  1. Two bilingual researchers independently  - A bilingual nursing professor  2. An independent bilingual translator  = Minor changes | Not reported | Experts (7)  = I-CVI^8^ and S-CVI^9^ both 0.95 | Nurses, charge nurses, doctors, pharmacists, and therapists (30) | Nurses (214),  charge nurses (82), and head nurses (6)  = 305  General wards, intensive care units, outpatient units,  and specialized  units | CFA^4^, the fit was assessed using the chi-square/degree of freedom, RMSEA^5^, CFI^6^, AIC^11^, TLI^13^. A factor loading > 0.40 was accepted, 7-factor models were tested.  = χ2 = 578.212, df 303,  p < 0.001, RMSEA^5^ 0.055, CFI^6^ 0.923, AIC^11^ 18,973.96 and TLI^13^ 0.904 | 1. Cronbach’s alpha and intraclass  correlation coefficients  = The total scale alpha 0.91. Cronbach’s alpha ranges between dimensions from 0.59 to 0.87. The lowest alpha in dimension Institutional support (0.59), Colleague support (0.63) and Professional self-efficacy (0.63).  The intraclass consistent coefficient was 0.71. | K-SVEST includes eight factors with a total of 28 items. |
| Knudsen, T., Abrahamsen, C., Jorgensen, J., & Schroder, K. (2022), Denmark,  D-SVEST | (Danish)  WHO’s guidelines  1. One author with the support of the original developers  2. One bilingual professor  = A few minor changes | Was conducted in connection with the pilot test, HCPs^1^ (3)  = Minor adjustments, the 5-point Likert scale sufficient | Not reported | HCPs^1^ (3)  = Minor adjustments | Midwives (74), physicians (67), nurses (25)  and nursing assistants (5)  = 171  Obstetrics and gynaecology,  internal medicine  and emergency | CFA^4^, the fit was assessed using RMSEA^5^, CFI^6^, SRMR^10^, and the chi-square test. A factor loading >0.40 was accepted, 7-factor models were tested.  = 21 items’ factor loading >0.40 and four items’ (9, 10, 15 and 25) factor loading <0.40.  Removing items 9, 10, 15 and 25 improved the model’s fit: x^2^ from 457.034 to 342.783, df from 254 to 188, p≥0.001 in both cases, RMSEA^5^ from 0.069 to 0.070, CFI^6^ from 0.884 to 0.908, and SRMR^10^ from 0.094 to 0.076).  In the end, all original items were kept. | 1. Cronbach’s alpha  = The total scale alpha 0.91. Cronbach’s alpha ranges between dimensions from 0.40 to 0.88.  The lowest alpha in dimensions Colleague support (0.40) and Institutional support (0.68).  Items 9, 10, 15 and 25 had low (<0.3) item-rest correlations. | D-SVEST includes seven factors with a total of items 25 items and two outcome variables.  Modifications to the wording of 9 (“I appreciate my co-workers’ attempts to console  me, but their efforts can come at the wrong time”) and 25 (“These situations do not make me question my  professional abilities”) items. |
| Koca, A., Elhal, A.H., Genç, S., Oǧuz, A.B., Eneyli, M.G., & Polat, O. (2022),  Turkey,  T-SVEST | (Turkish)  WHO’s guidelines  1. Two bilingual emergency medicine medical doctors  - Experts (5)  2. Two additional bilingual medical doctors | Not reported | Experts (10)  = I-CVI^8^ values >0.80 and S-CVI/Ave^14^ 0.92 | HCPs^1^ (10) | Physicians (92), residents (80),  nurses (38),  medical doctors (11)  = 221  Emergency department | Bartlett’s sphericity test and KMO^3^  = KMO^3^ 0.84, Bartlett’s test p<0.001  CFA^4^, the fit was assessed using the Chi-square test, RMSEA^5^, CFI^6^, and TLI^13^. A factor loading >0.40 was accepted, 7-factor models were tested.  = p<0.001, RMSEA^5^ 0.071, CFI^6^ 0.931, AIC^11^625.422, TLI^13^ 0.911. A good fit for a nine-factor structure and 24 items | 1. Cronbach’s alpha  = The total scale alpha 0.90. Cronbach’s alpha ranges between dimensions from 0.78 to 0.89. The lowest alpha in dimension Colleague support. | T-SVEST includes 9 factors with a total of 24 items. |
| Pieretti, A., Bastiani, L., Bellando, T., Molinaro, S., Zoppi., P., & Rasero, L. (2022),  Italia,  I-SVEST | (Italian)  Beaton et al.’s (2000) guidelines.  1. Two translators independently  2. A native speaker | Not reported | Not reported | Not conducted | Nurses (56),  doctors (29)  = 85  Surgical, intensive care, and internal medicine | PCA^15^ with orthogonal rotation (varimax method). Eigenvalue rule (number of factors with eigenvalue >1), and factor loading rule (item-factor correlations  >0.30, suggested for behavioural phenotypes interpretation) were used.  = Identified 7 principal component  with eigenvalue >1  CFA^4^, the fit was assessed using RMSEA^5^, CFI^6^, and SRMR^10^.  = χ2 = 88.46, df = 68, p< 0.051, RMSEA^5^ 0.075, CFI^6^ 0.91, and SRMR^10^ 0.091. | 1. Cronbach’s alpha  = The total scale alpha 0.855. Cronbach’s alpha ranges between dimensions from 0.613 to 0.885. The lowest alpha in dimension Colleague support (0.613) and Absenteeism (0.694). | I-SVEST includes 7 factors with a total of 25 items. |
| Santana-Domínguez, I., González-de la Torre, H., Verdú-Soriano, J., Nolasco, A., & Martín-Martínez, A. (2022),  Spain,  SVEST-E | (Spanish)  The translation process presented in Santana-Domínguez et al.’s (2021) article:  1. Two translators independently  - The research team, a gynaecologist and two midwives  2. Two bilingual translators independently  - The research team and one original author. | Not reported | Presented in Santana-Domínguez et al.’s (2021) article:  The expert panel (10)  = I-CVI^8^ range 0.6-1 | Obstetricians and midwives (21) | Midwives (366), obstetrician physicians (323)  = 689  Professional work with direct care to women (for example, obstetrics and gynaecology) | EFA^2^ with a polychoric correlation matrix with factor extraction by unweighted least squares and PROMIN rotation. EFA’s fit was assessed using the KMO^3^ and Bartlett sphericity test. A factor loading >0.30 was accepted, 7-factor models were tested. A random sample of 360 participants of the total 689 participants.  = KMO^3^ 0.86, Bartlett’s test p=0.00001, RMSEA^5^ 0.038, and CFI^6^ 0.989. All items had factor loading >0.30 in the assigned factor, except item “My supervisor blames the team members when these cases occur”.  CFA^4^ with a matrix of semispecified factor loading coefficients.  The rest of 329 participants.  = KMO^3^ 0.85, Bartlett’s test p=0.00001, RMSEA^5^ 0.038, and CFI^6^ 0.989. | 1. The ORION coefficients, the global reliability using the McDonald’s omega coefficient.  = The ORION coefficients 0.92. The McDonald’s omega coefficient 0.88. | SVEST-E includes five factors with a total of 36 items. |
| Sharif-Nia, H. & Hanifi, N. (2022), Irak,  P-SVEST | (Persian)  Beaton et al. (2002) guideline  1. Two English–Persian translators independently  2. A Persian–English translator | Nurses (20) | Faculty  members from the Department of intensive care nursing and emergency nursing (6) and nursing managers (4)  = I-CVI^8^ range 0.8-1 | Not conducted | Nurses (754)  = 754  Coronary Care Unit, Emergency Department, Intensive Care Unit, Neonatal Intensive Care Unit,  Paediatric Intensive Care Unit, and Haemodialysis | EFA^2^ with Promax rotation,  KMO^3^, and Bartlett’s test of sphericity. Eigenvalue rule (number of factors with eigenvalue >1). A factor loading >0.30 was accepted. A random sample of 377 participants of a total of 754 participants.  = KMO^3^ 0.830, Bartlett’s test p < 0.001. 14 items were removed based on factor loading <0.3 and communalities <0.2. Four factors were extracted.  CFA^4^, the fit was assessed using the Chi-square test, Chi-square/degree of freedom, RMSEA^5^, CFI^6^, SRMR^10^, TLI^13^, GFI^16^, NFI^17^, RFI^18^, IFI^19^,  The rest of the 377 participants.  =χ2 (81) = 1630.195,  p < 0.001, χ2 /df = 2.015, RMSEA^5^ 0.058, CFI^6^ 0.956, TLI^13^ 0.944, GFI^16^ 0.932, NFI^17^ 0.918, and  IFI^19^ 0.957. | 1. Cronbach’s  alpha, McDonald’s omega  =  Cronbach’s  alpha ranges between dimensions from 0.702 to 0.869.  Omega ranges between dimensions from 0.705 to 0.875.  2. Cronbach's alpha and McDonald's omega, composite reliability, and maximum reliability  = for all factors >0.7  3. The convergent and divergent validity: Fornell and Larcker's approach (1981).  4. Multivariate normality: Mardia's coefficient of multivariate kurtosis,  Mardia's coefficient and Mahalanobis distance | P-SVEST includes four factors with a total of 15 items. |
| Strametz, R., Siebold, B., Heistermann, P., Haller, S., & Bushuven, S. (2022),  Germany,  G-SVEST-R | (German)  WHO’s guideline  1. *not clear*  2. One native English speaker not familiar with the original scale | Expert panel’s (four physicians) evaluation after translation process.  The pilot test’s participants’ interviews, nurses, and physicians (10)  = Minor revision to two items | Not reported | Nurses and physicians (10) | Physician assistants (83), nurses (75), physicians (56), paramedics (52), medical assistants (24), medical therapists (15), remedial therapists (1)  = 306  Many different medical disciplines | PCA^15^, the fit was assessed using The Bartlett sphericity test and KMO^3^. Varimax rotation and eigenvalues were used.  = p<0.001, KMO^3^ 0.836 | 1. Cronbach’s alpha and Guttman criteria  = The total scale alpha 0.884. *Cronbach’s alpha ranges between dimensions are not mentioned.*  A very high Guttmans lambda-2 of 0.9, and a high lambda-4 of 0.834. | G-SVEST-R includes 11 factors with a total of 42 items. The issue with item 31 (“When I am at work, I am distracted and not 100% present because of my involvement in these situations”) must be taken into account in future research using the G-SVESTR. |
| Thungjaroenkul, P., Soivong, P., Udkanta, K., & Tiansawad, S. (2024). Thailand.  Thai-SVEST-R | (Thai)  WHO’s guideline  1) Forward translation by two bilingual researchers  2) Expert panel review  3) Back translation by two other bilingual translators  4) pre-testing and cognitive interviewing | Not reported | Not reported | Not reported | Nurses (400) | CFA^4^  values in all fit indices (λ2  = 1,010.63; df = 51; λ2 /df = 1.98; CFI = .91; RMSEA  = .04). | Cronbach’s alpha coefficients ranged from .73 to .92, | The results found that Part A comprised 35 items that are psychosocial and employment-related factors including nine dimensions: psychological distress, physical distress, colleague support, supervisor support, institutional support, professional self-efficacy, absenteeism, turnover intentions, and resilience dimensions as same as the English version of SVEST-R. All items had factor loadings above the conventional loading level of .40, ranging from .52 to .94. |
| Zhang, X., Chen, J., & Lee, S-Y. (2021),  China,  C-SVEST | (Mandarin Chinese)  Brislin’s (1970) guidelines  1. Two bilingual researchers independently  - A bilingual nursing professor translation  2. Two independent bilingual translators  - One SVEST scale’s original developer | Not reported | Professional experts (6)  = I-CVI^8^ range 0.85-0.97 and S-CVI^9^ 0.91,  some terms clarified | Not conducted | Nurses (625)  = 625  Internal medicine, surgery, emergency, intensive and other units | EFA^2^ with KMO^3^, and the Bartlett test. Eigenvalue >1, factor loading of >0.5. A random sample of 358 participants out of a total of 625 participants  = KMO^3^ 0.82, the Bartlett test p<0.001. Eight items were removed.  CFA^4^ using AMOS for maximum likelihood estimation, the fit was assessed using the chi-square test, the chi-square/degree of freedom, RMSEA^5^, CFI^6^, TLI^13^, GFI^16^, NFI^17^, Parsimony goodness fit index. 6-factor model was used. The rest of the 267 participants.  = χ2/df = 2.19, RMSEA^5^ 0.07, CFI^6^, 0.90, GFI^16^ 0.84. A good fit for a nine-factor structure | 1. Cronbach’s alpha and Spearman-Brown coefficient  = The total scale alpha *not reported*. Cronbach’s alpha ranges between dimensions from 0.59 to 0.92. The lowest alpha in the dimension of Colleague support (0.59), Institutional support (0.60), and Professional self-efficacy (0.61).  Spearman-Brown coefficient 0.88. | The C-SVEST includes nine factors with a total of 29 items. |
| Winning, A.M., Merandi, J., Rausch, J.R., Liao, N., Hoffman, J.M., Burlison, J.D., & Gerhardt, C.A. (2021),  USA,  SVEST-R (Revised version of the SVEST) | The SVEST-R is in American English and developed based on the original SVEST, literature and the experts’ (two RNs, two physicians, a pharmacist, and a social worker) suggestions. 14 items were added to the original SVEST. | Not reported | Not reported | Not conducted | RN (223),  physician assistants/nurse practitioners (34), respiratory therapists (13), physicians (9), pharmacists (8), physical/  occupational therapists (7),  unit assistants (6), patient care assistants (4),  licensed practical nurses (3), management (2), dieticians (1), technicians (1), and others (5)  = 514  Neonatal ICU | CFA^4^, the fit was assessed using the Chi-square test, RMSEA^5^, CFI^6^, and SRMR^10^.  = Eight items were removed based on low factor loading. χ2 = 1555.6, df = 524, p< 0.0001, RMSEA^5^ 0.079, CFI^6^ 0.821,  SRMR^10^ 0.091. | 1. Cronbach’s alpha  = The total scale alpha *not reported*. Cronbach’s alpha ranges between dimensions from 0.66 to 0.86. The lowest alpha in dimensions Colleague support. | The SVEST-R includes nine factors with a total of 35 items. |

^1^ = Healthcare professionals, ^2^ = Exploratory factor analysis, ^3^ = The Kaiser-Meyer-Olkin test, ^4^ = Confirmatory factor analysis, ^5^ = The root mean square error of approximation, ^6^ = Comparative fit index, ^7^ = Content validity ratio, ^8^ = Item-level content validity index, ^9^ = Scale-level content validity index, ^10^ = The standardised root mean squared residual, ^11^ = Akaike information criterion, ^12^ = Bayesian information criterion, ^13^ = Tucker–Lewis index, ^14^ = Scale-level content validity index average, ^15^ = Principal component analysis, ^16^ = Goodness-of-fit index, ^17^ = Normed fit index, ^18^ = Relative fit index, ^19^ = Incremental fit index

**Appendix 2.** Available SVEST and SVEST-R scales and the model fit testing results of the translated scales where CFA has been conducted

| **Authors, (year),**  **Country,**  **SVEST or**  **SVEST-R** | **Number of factors/ items** | **χ2** | **df; x2/df*** | **Probability level** | **CFI **** | **TLI**** | **RMSEA** | **SRMR** |
| --- | --- | --- | --- | --- | --- | --- | --- | --- |
| Kamaruzaman et al., (2022), Malaysia,  M-SVEST-R | 7/32 | 797 | 418; - | < 0.0001 | .946 | .935 | .051 | .055 |
| Strametz et al., (2022), Germany  G-SVEST-R | 11/42 | n/a | n/a | n/a | n/a | n/a | n/a | n/a |
| Thungjaroenkul et al., (2023). Thailand.  Thai-SVEST-R | 9/35 | 1,010.63 | 51; 1.98 | ? | .910 | - | .040 | - |
| Winning et al., (2021), USA, SVEST-R | 9/35 | 1555.600 | 524; - | < 0.0001 | .821 | - | 0.079 | .091 |
| Brunelli et al. (2021), Argentina  SVEST | 7/26 | n/a | n/a | n/a | n/a | n/a | n/a | n/a |
| Burlison et al. (2017), USA,  The original SVEST | 7/29 | 566.06 | 254; - | < 0.01 | .910 | - | .066 | - |
| De Sordi et al. (2022),  Brazil,  BR-SVEST | 9/36 | n/a | n/a | n/a | n/a | n/a | n/a | n/a |
| Kim et al. (2020),  South Korea,  K-SVEST | 8/28 | 578.212 | 303; - | < 0.001 | .923 | 904 | 904 | - |
| Knudsen et al.(2022), Denmark,  D-SVEST | 7/25 | 342.783 | 188; - | <0.001 | .908 | - | .070 | 076 |
| Koca et al. (2022),  Turkey,  T-SVEST | 9/24 |  | - ; 2.120 | <0.001 | .931 | .911 | .071 | - |
| Pieretti et al., (2022), Italia,  I-SVEST | 7/25 | 88.46, | 68; - | < 0.051 | .910 | - | .075 | .091 |
| Santana-Domínguez et al., (2022),  Spain,  SVEST-E | 5/36 |  | - | - | .989 | - | .038 | - |
| Scarpis et al., (2022), Italy, IT-SVEST | 9/29 | 676.18 | 327; - | < 0.001 | 0.930 | 0.913 | 0.055 | - |
| Sharif-Nia & Hanifi (2022), Irak,  P-SVEST | 11/42 | 1630.195 | 81; - | < 0.001 | .956 | .944 | .058 | - |
| Zhang et al. (2021),  China,  C-SVEST | 9/29 | - | - ; 2.1 | - | .900 | - | .070 | - |

Note: The upper part of the table presents SVEST-R versions and the lower part SVEST versions.

- Target value: x2/df* (cutoff ≥ 2 or 3) (Schreiber et al., 2006).

** Target values: CFI a cutoff value close to .95, (rejection if CFI<.95 or <.96), TLI a cutoff value close to .95, SRMR <.05 close fit, .05-.08 fair fit, >.10 poor fit a cutoff value close to .08 (rejection if SRMR>.08), RMSEA a cutoff value close to .06 (rejection if RMSEA >.06 (Hu & Bentler, 1999).

**Appendix 3.** Covariances

| Items |  | Factors | Estimate | S.E. | C.R. | P |
| --- | --- | --- | --- | --- | --- | --- |
| F1 | <--> | F2 | ,695 | ,123 | 5,635 | *** |
| F1 | <--> | F3 | ,185 | ,062 | 2,984 | ,003 |
| F1 | <--> | F4 | ,183 | ,085 | 2,151 | ,031 |
| F1 | <--> | F5 | ,226 | ,098 | 2,311 | ,021 |
| F1 | <--> | F6 | ,735 | ,127 | 5,777 | *** |
| F1 | <--> | F7 | ,550 | ,113 | 4,861 | *** |
| F1 | <--> | F8 | ,277 | ,088 | 3,163 | ,002 |
| F1 | <--> | F9 | -,193 | ,065 | -2,966 | ,003 |
| F2 | <--> | F3 | ,278 | ,074 | 3,777 | *** |
| F2 | <--> | F4 | ,339 | ,096 | 3,523 | *** |
| F2 | <--> | F5 | ,281 | ,107 | 2,634 | ,008 |
| F2 | <--> | F6 | ,753 | ,132 | 5,696 | *** |
| F2 | <--> | F7 | ,739 | ,130 | 5,689 | *** |
| F2 | <--> | F8 | ,382 | ,097 | 3,926 | *** |
| F2 | <--> | F9 | -,029 | ,066 | -,440 | ,660 |
| F3 | <--> | F4 | ,370 | ,078 | 4,742 | *** |
| F3 | <--> | F5 | ,041 | ,061 | ,661 | ,509 |
| F3 | <--> | F6 | ,329 | ,080 | 4,089 | *** |
| F3 | <--> | F7 | ,277 | ,075 | 3,703 | *** |
| F3 | <--> | F8 | ,244 | ,065 | 3,758 | *** |
| F3 | <--> | F9 | ,003 | ,039 | ,078 | ,938 |
| F4 | <--> | F5 | ,227 | ,096 | 2,355 | ,019 |
| F4 | <--> | F6 | ,386 | ,102 | 3,789 | *** |
| F4 | <--> | F7 | ,459 | ,104 | 4,436 | *** |
| F4 | <--> | F8 | ,287 | ,086 | 3,348 | *** |
| F4 | <--> | F9 | ,051 | ,060 | ,850 | ,395 |
| F5 | <--> | F6 | ,273 | ,112 | 2,442 | ,015 |
| F5 | <--> | F7 | ,486 | ,118 | 4,110 | *** |
| F5 | <--> | F8 | ,080 | ,093 | ,855 | ,393 |
| F5 | <--> | F9 | ,091 | ,069 | 1,316 | ,188 |
| F6 | <--> | F7 | ,788 | ,134 | 5,861 | *** |
| F6 | <--> | F8 | ,392 | ,101 | 3,869 | *** |
| F6 | <--> | F9 | -,084 | ,070 | -1,202 | ,230 |
| F7 | <--> | F8 | ,652 | ,111 | 5,863 | *** |
| F7 | <--> | F9 | ,074 | ,069 | 1,070 | ,285 |
| F8 | <--> | F9 | ,028 | ,060 | ,474 | ,636 |
| e34 | <--> | e35 | ,299 | ,081 | 3,693 | *** |
| e26 | <--> | e28 | -,161 | ,036 | -4,444 | *** |
| Items |  | Factors | Estimate | S.E. | C.R. | P |

*Note:* The acronym explanations are S.E. = Standard Error, C.R. = xx
